# Supplementary material for: Gene expression-based identification of prognostic markers in lung adenocarcinoma
Source: PLoS One. 2025 May 7;20(5):e0310232. doi: 10.1371/journal.pone.0310232 (PMC12057878; doi:10.1371/journal.pone.0310232)
Supplement: S1 Table — (DOCX) [file pone.0310232.s001.docx]

| Antigen | Clone | Dilution | Pretreatment | Staining System | Control Tissue |
| --- | --- | --- | --- | --- | --- |
| Ki67 | MIB1 | 1:200 | Dako high pH | Dako Autostainer+, EnVision | Tonsil |
| MCM4 | D3H6N | 1:200 | Dako high pH | Dako Autostainer+, EnVision | Tonsil |
| TYMS | EPR4545 | 1:50 | CC1 | Ventana Discovery Ultra | Tonsil |

**Supplementary Table 1.** Immunohistochemical staining for Ki67, MCM4 and TYMS.
