# Supplementary material for: Gene expression-based identification of prognostic markers in lung adenocarcinoma
Source: PLoS One. 2025 May 7;20(5):e0310232. doi: 10.1371/journal.pone.0310232 (PMC12057878; doi:10.1371/journal.pone.0310232)
Supplement: S2 Table — (DOCX) [file pone.0310232.s002.docx]

**Supplementary Table 2**. Multivariate Cox regression analysis for 5-year overall survival for Ki67, MCM4, and TYMS protein expression in the IHC validation cohort.

|  | **5-year overall survival** | |
| --- | --- | --- |
| **Variable** | **HR (95% CI)** | ***P*-value** |
| **KI67 PROTEIN EXPRESSION** |  |  |
| Low | Reference |  |
| High | 1.29 (0.68-2.45) | *P* = 0.43 |
| **Stage** |  |  |
| 1 | Reference |  |
| 2 | 4.08 (1.92-8.7) | *P* < 0.01 |
| 3 | 4.24 (1.94-9.27) | *P* < 0.01 |
| 4 | NA | NA |
| **Growth pattern** |  |  |
| Predominant acinary/papillary | Reference |  |
| Mucinous or predominant micropapillary/solid | 1.56 (0.88-2.75) | *P* = 0.12 |
| Minimally invasive/predominant lepidic | 0 (0-Inf) | *P* = 0.996 |
| **Gender** |  |  |
| Male | Reference |  |
| Female | 1.17 (0.64-2.11) | *P* = 0.61 |
| **Age** |  |  |
| Years (as numeric) | 1.01 (0.97-1.05) | *P* = 0.74 |
| **Smoking** |  |  |
| Current | Reference |  |
| Former | 0.72 (0.4-1.28) | *P* = 0.26 |
| Never | 0.18 (0.04-0.81) | *P* = 0.02 |
| **WHO performance status** |  |  |
| 0 | Reference |  |
| 1 | 1.34 (0.79-2.28) | *P* = 0.28 |
| 2 | 2.34 (0.5-10.86) | *P* = 0.28 |
| **Adjuvant treatment** |  |  |
| No | Reference |  |
| Yes | 0.61 (0.34-1.12) | *P* = 0.11 |
| **MCM4 protein expression** |  |  |
| Low | Reference |  |
| High | 1.11 (0.5-2.44) | *P* = 0.80 |
| **Stage** |  |  |
| 1 | Reference |  |
| 2 | 3.77 (1.87-7.59) | *P* =< 0.01 |
| 3 | 4.37 (2.14-8.9) | *P* = < 0.01 |
| 4 | NA | NA |
| **Growth pattern** |  |  |
| Predominant acinary/papillary | Reference |  |
| Mucinous or predominant micropapillary/solid | 1.59 (0.96-2.63) | *P* = 0.07 |
| Minimally invasive/predominant lepidic | 0 (0-Inf) | *P* = 0.996 |
| **Gender** |  |  |
| Male | Reference |  |
| Female | 0.99 (0.58-1.69) | *P* = 0.98 |
| **Age** |  |  |
| Years (as numeric) | 1 (0.97-1.04) | *P* = 0.90 |
| **Smoking** |  |  |
| Current | Reference |  |
| Former | 0.72 (0.43-1.21) | *P* = 0.22 |
| Never | 0.27 (0.09-0.8) | *P* = 0.02 |
| **WHO performance status** |  |  |
| 0 | Reference |  |
| 1 | 1.4 (0.85-2.29) | *P* = 0.19 |
| 2 | 2.7 (0.6-12.04) | *P* = 0.19 |
| **Adjuvant treatment** |  |  |
| No | Reference |  |
| Yes | 0.65 (0.36-1.16) | *P* = 0.14 |
| **TYMS protein expression** |  |  |
| Low | Reference |  |
| High | 1.14 (0.55-2.37) | *P* = 0.73 |
| **Stage** |  |  |
| 1 | Reference |  |
| 2 | 4.15 (1.74-9.91) | *P* =< 0.01 |
| 3 | 3.83 (1.74-8.45) | *P* =< 0.01 |
| 4 | NA | NA |
| **Growth pattern** |  |  |
| Predominant acinary/papillary | Reference |  |
| Mucinous or predominant micropapillary/solid | 1.86 (1.01-3.42) | *P* = 0.05 |
| Minimally invasive/predominant lepidic | 0 (0-Inf) | *P* = 0.996 |
| **Gender** |  |  |
| Male | Reference |  |
| Female | 1.2 (0.65-2.21) | *P* = 0.56 |
| **Age** |  |  |
| Years (as numeric) | 1.01 (0.97-1.06) | *P* = 0.64 |
| **Smoking** |  |  |
| Current | Reference |  |
| Former | 0.73 (0.41-1.33) | *P* = 0.30 |
| Never | 0.16 (0.04-0.71) | *P* = 0.02 |
| **WHO performance status** |  |  |
| 0 | Reference |  |
| 1 | 1.25 (0.71-2.18) | *P* = 0.44 |
| 2 | 2.27 (0.5-10.29) | *P* = 0.29 |
| **Adjuvant treatment** |  |  |
| No | Reference |  |
| Yes | 0.61 (0.3-1.21) | *P* = 0.16 |
