# Supplementary material for: Gene expression-based identification of prognostic markers in lung adenocarcinoma
Source: PLoS One. 2025 May 7;20(5):e0310232. doi: 10.1371/journal.pone.0310232 (PMC12057878; doi:10.1371/journal.pone.0310232)
Supplement: S1 Fig — (PDF) [file pone.0310232.s003.pdf]

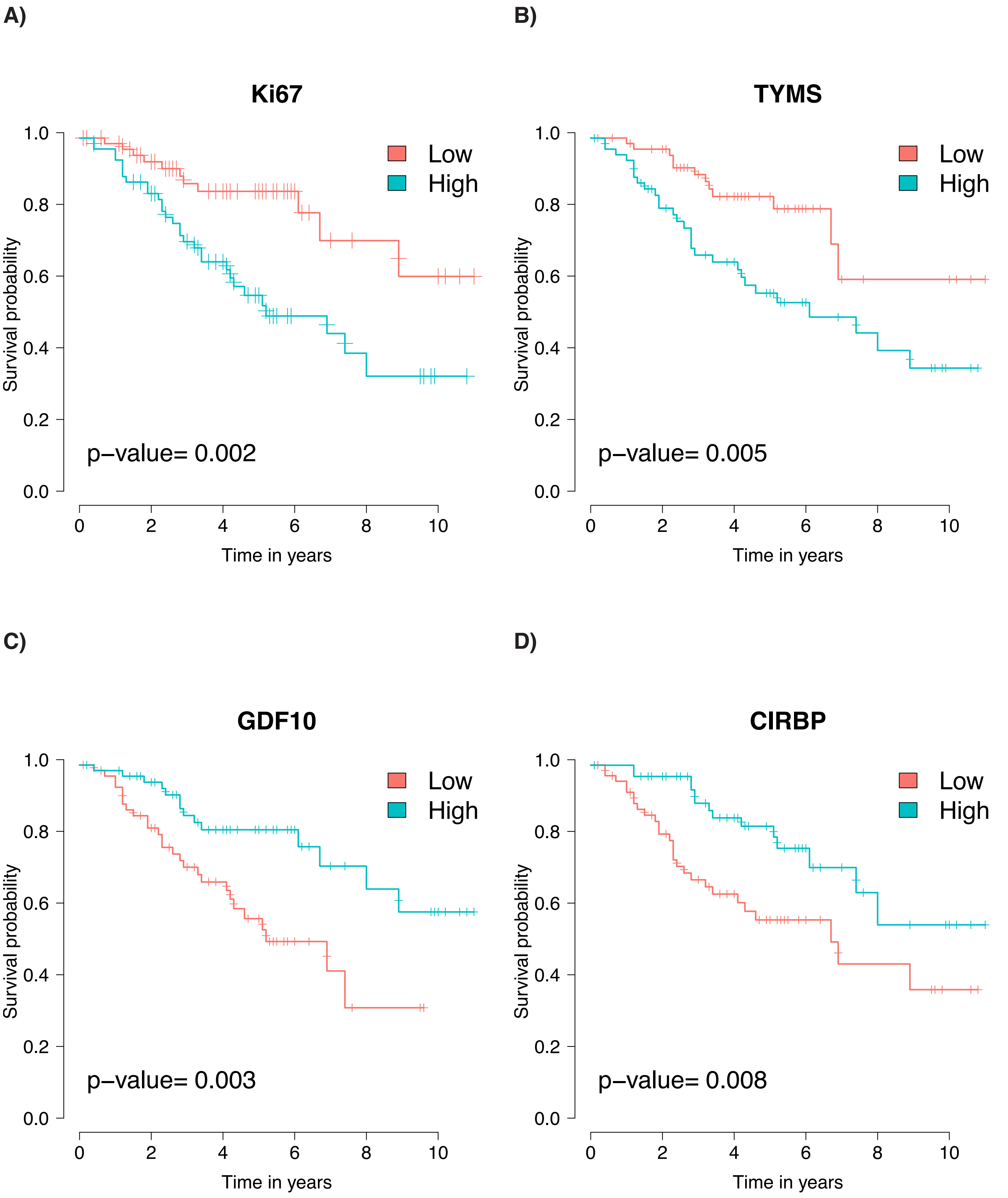

Supplementary Figure S1. Prognostic value of Ki67 (*MKI67*) (A), *TYMS* (B), *GDF10* (C), and *CIRBP* (D) gene expression levels in one of the validation data set using overall survival as clinical endpoint (Tang et al.14). P-values calculated using the log-rank test using full follow-up data.
