# Supplementary material for: Gene expression-based identification of prognostic markers in lung adenocarcinoma
Source: PLoS One. 2025 May 7;20(5):e0310232. doi: 10.1371/journal.pone.0310232 (PMC12057878; doi:10.1371/journal.pone.0310232)
Supplement: S2 Fig — (A) Tomida et al. [15], (B) Tang et al. [14]. (PDF) [file pone.0310232.s004.pdf]

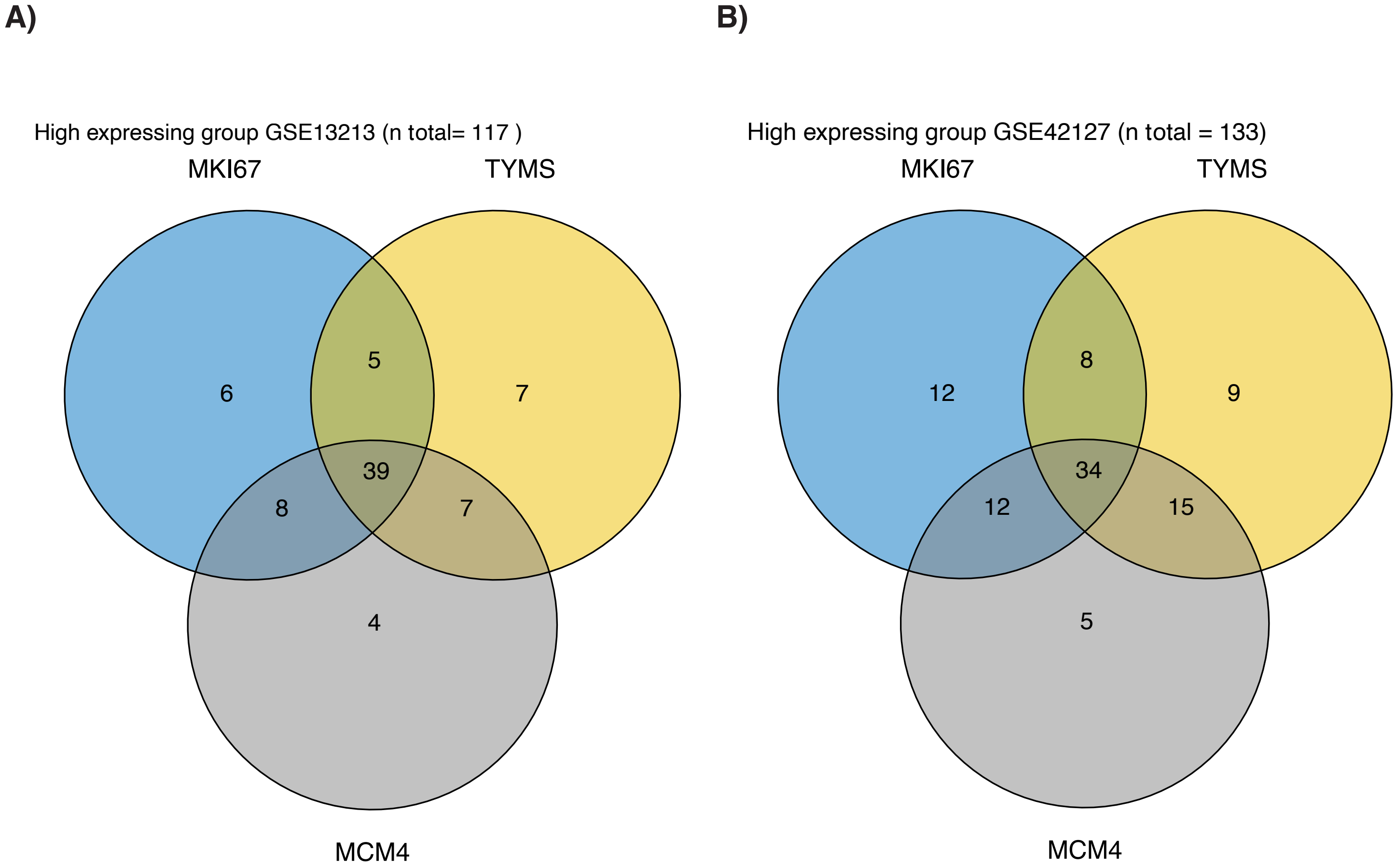

Supplementary Figure S2. The overlap between cases with high gene expression levels (cut-off based on the median gene expression values for each gene) of *Ki67*, *TYMS*, and *MCM4* in the two validation data sets. (A) Tomida et al. ref 15 (GSE13213), (B) Tang et al. ref 14 (GSE42127).
