# Supplementary material for: Gene expression-based identification of prognostic markers in lung adenocarcinoma
Source: PLoS One. 2025 May 7;20(5):e0310232. doi: 10.1371/journal.pone.0310232 (PMC12057878; doi:10.1371/journal.pone.0310232)
Supplement: S3 Fig — For Ki67 and MCM4, nuclear staining was considered positive. For TYMS, cytoplasmic or nuclear staining were considered positive, although only cells with visible nuclei were counted. (A) Low expression of Ki67. (B) High expression of Ki67. (C) Low expression of MCM4. (D) High expression of MCM4. (E) Low expression of TYMS. (F) High expression of TYMS. (DOCX) [file pone.0310232.s005.docx]

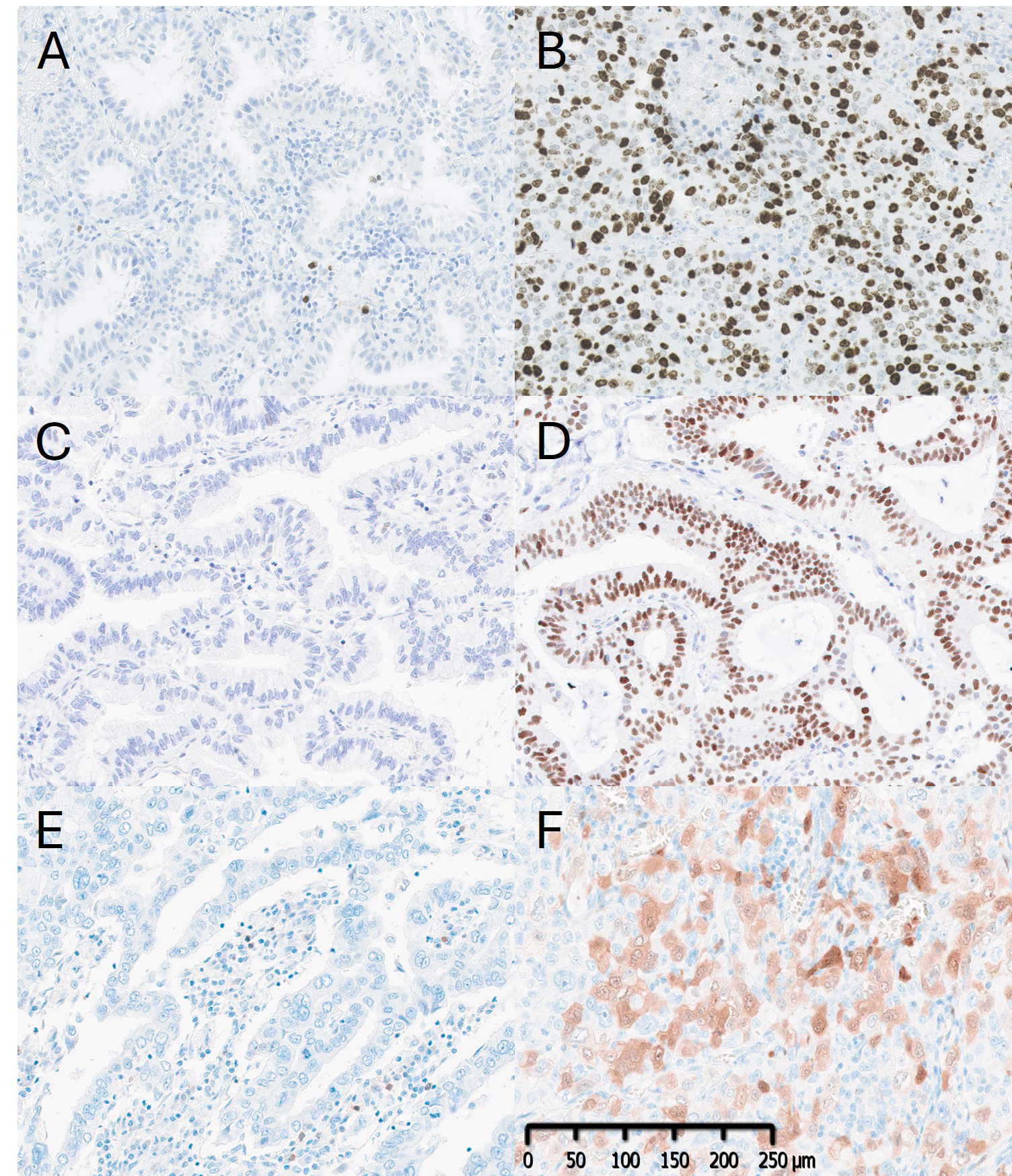


Supplementary Figure 3. Representative microscopic images of the stainings. For Ki67 and MCM4, nuclear staining was considered positive. For TYMS, cytoplasmic or nuclear staining were considered positive, although only cells with visible nuclei were counted. (A) Low expression of Ki67. (B) High expression of Ki67. (C) Low expression of MCM4. (D) High expression of MCM4. (E) Low expression of TYMS. (F) High expression of TYMS**.**
