# Supplementary material for: Gene expression-based identification of prognostic markers in lung adenocarcinoma
Source: PLoS One. 2025 May 7;20(5):e0310232. doi: 10.1371/journal.pone.0310232 (PMC12057878; doi:10.1371/journal.pone.0310232)
Supplement: S4 Fig — (PDF) [file pone.0310232.s006.pdf]

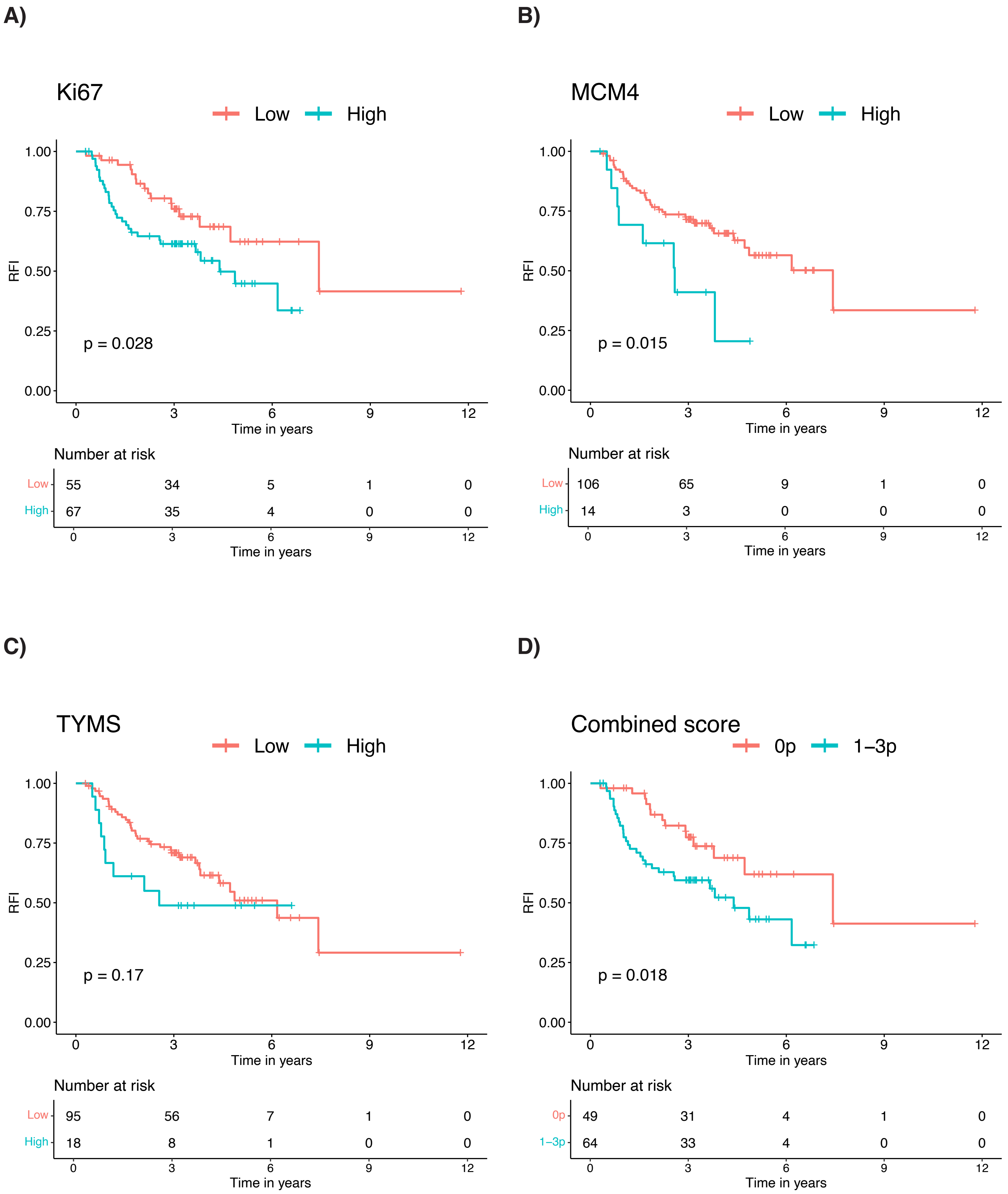

Supplementary Figure S4. Prognostic value of Ki67 (A), MCM4 (B), TYMS (C), and combined score (D), on recurrence-free interval (RFI) in the IHC discovery cohort. P-values calculated using the log-rank test using full follow-up data.
