# Supplementary material for: Gene expression-based identification of prognostic markers in lung adenocarcinoma
Source: PLoS One. 2025 May 7;20(5):e0310232. doi: 10.1371/journal.pone.0310232 (PMC12057878; doi:10.1371/journal.pone.0310232)
Supplement: S6 Fig — (DOCX) [file pone.0310232.s008.docx]

TYMS


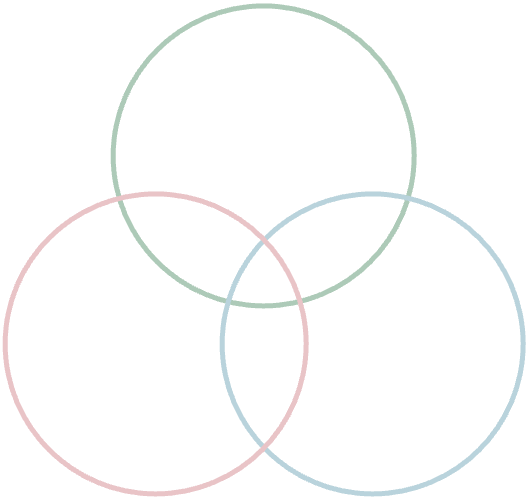


Ki67

0

5

6

12

2

0

MCM4

44

TYMS


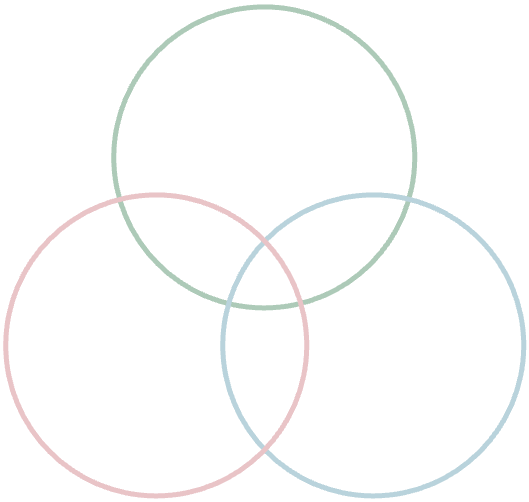


Ki67

0

5

3

10

1

0

MCM4

59

A)

B)

Supplementary Figure 6. The overlap between cases that were positive for the three markers in the IHC discovery cohort (A) and the IHC validation cohort (B).
