# Supplementary material for: Gene expression-based identification of prognostic markers in lung adenocarcinoma
Source: PLoS One. 2025 May 7;20(5):e0310232. doi: 10.1371/journal.pone.0310232 (PMC12057878; doi:10.1371/journal.pone.0310232)
Supplement: S7 Fig — (PDF) [file pone.0310232.s009.pdf]

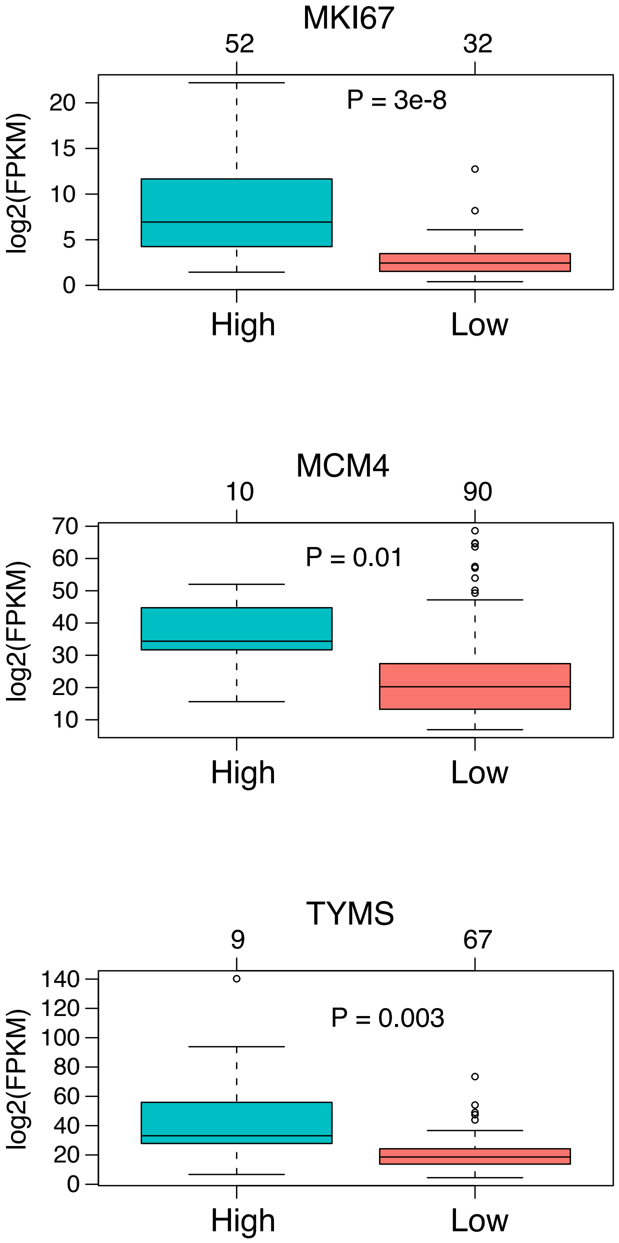

Supplementary Figure S7. The association between gene expression levels (y-axis, log2 FPKM levels) and immunohistochemical classification (x-axis, low or high expression) for the three markers in the IHC validation cohort. Two-sided p-values calculated using Wilcoxon's test.
