# Supplementary material for: Gene expression-based identification of prognostic markers in lung adenocarcinoma
Source: PLoS One. 2025 May 7;20(5):e0310232. doi: 10.1371/journal.pone.0310232 (PMC12057878; doi:10.1371/journal.pone.0310232)
Supplement: S8 Fig — (PDF) [file pone.0310232.s010.pdf]

A)

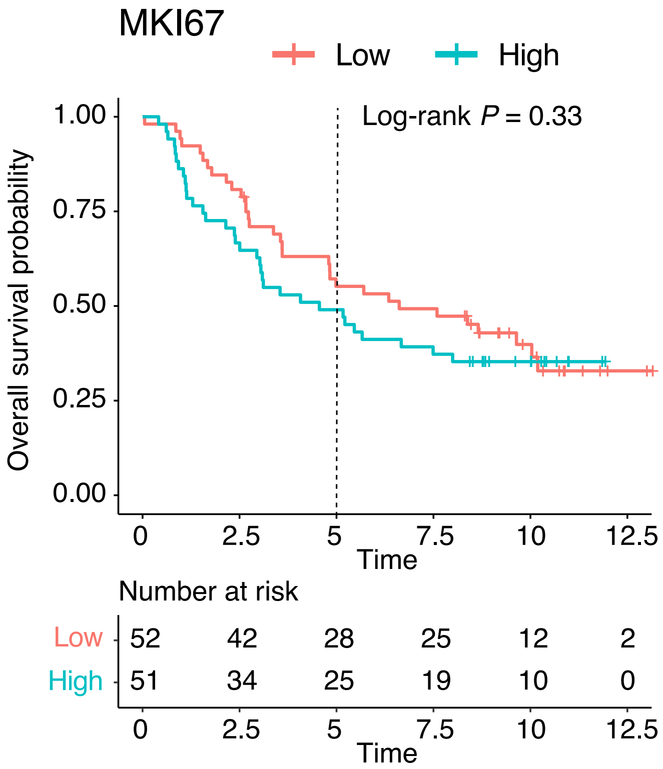

B)

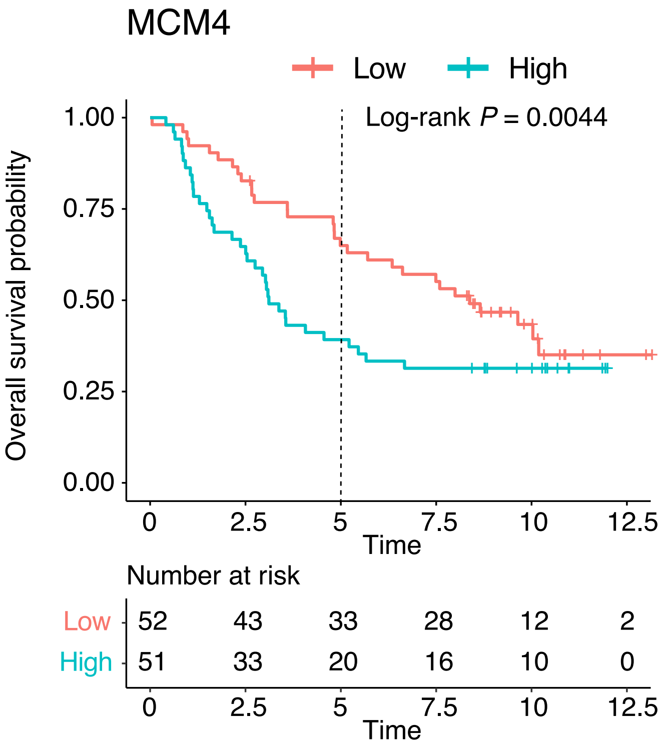

C)

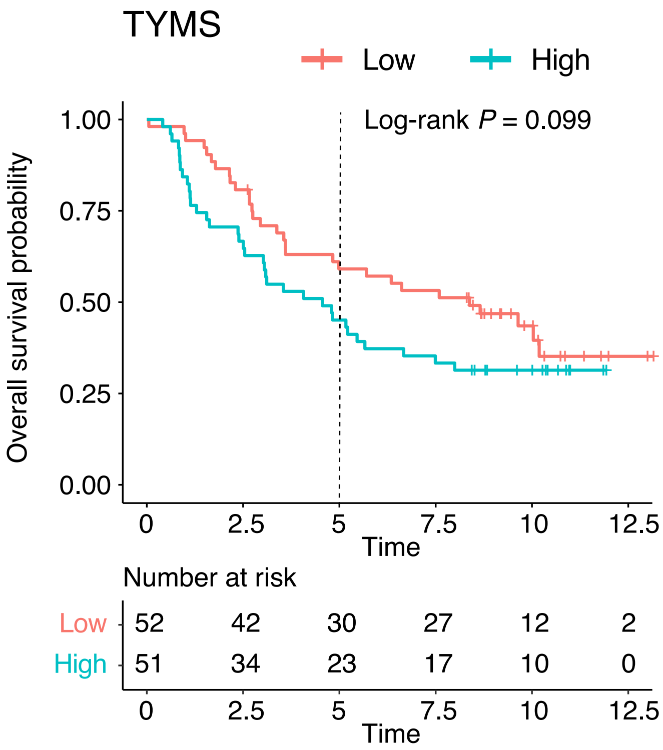

Supplementary Figure S8. The prognostic value of *Ki67* (*MKI67*) (A), *MCM4* (B), and *TYMS* (C) gene expression levels in the IHC validation cohort using overall survival as clinical endpoint. P-values calculated using the log-rank test for 5-year censored data. Samples with available data were split into two equally sized groups based on the respective gene expression values.
