# Supplementary material for: Gene expression-based identification of prognostic markers in lung adenocarcinoma
Source: PLoS One. 2025 May 7;20(5):e0310232. doi: 10.1371/journal.pone.0310232 (PMC12057878; doi:10.1371/journal.pone.0310232)
Supplement: Attachment — Submitted filename: PLOS ONE Review.docx [file pone.0310232.s011.docx]

The authors use publicly available microarray datasets to identify genes whose expression is associated with OS differences in lung adenocarcinoma and then tests clinical utility of select genes using IHC in Swedish cohorts. While correct statistical analysis was used and all conclusions are supported by the data, I had some minor concerns where further details or clarification are needed.

**Major**

- Methods
- Page 5, Figure 1
  - The authors may need to perform QC and additional filtering on microarray datasets. For example, Shedden et al. only retained array data for 442 of the 486 samples due to poor array quality (11 samples) or due to violations of clinical/pathological data (33 samples).
  - Additionally, the number of samples don’t always align with those reported in the original publications. Why did the authors only include 102 of the 193 samples from Chitale et al?
  - Similarly, how did you end up with 226 samples from Yamauchi et al? Was only the NCC-Tokyo cohort used from this publication?
    - The earliest publication associated with the GSE31210 dataset is Okayama et al. Cancer Research (2012) and they clearly state microarray data was generated for 226 cases. I would recommend either citing Okayama et al or both references for this dataset.
  - For improved clarity, it may be worthwhile to list the GEO accession ID for each of the microarray datasets.
- Page 9, Line 112
  - Could the authors please provide the rationale for including tertile split survival groups in addition to the median split survival groups?
    - It is not advised to use multiple different cutpoints unless it can be justified as it can appear to be a data “fishing” exercise, especially when the median cutpoint has been used consistently for all other gene expression analyses.
- Results
  - Page 15
    - Could the authors please include forest plots for the Cox proportional models shown in Figure 4? This would confirm the relationship between established prognostic factors (such as stage) and OS are as expected in this cohort.
  - Page 17, Line 218
    - I would recommend acknowledging that gene expression analysis of the IHC validation cohort was carried out using RNAseq rather than microarray as for the gene expression discovery and validation cohorts. This could also be mentioned in the discussion as technical differences between the platforms may have contributed to the results.

**Minor**

- Figures
  - Page 11, Figure 2
    - Please update the figure legend to include details such as what the size of circles indicates.
  - Page 28, Supplementary Figure 3
    - Please add scale bars to all IHC images. Additionally, so that figure can be understood on its own, the figure legend should include information about the criteria for positive staining (e.g. nuclear or nuclear and cytoplasmic).
  - Page 31, Supplementary Figure 7
    - Can the authors confirm what expression units for gene expression levels (e.g. logCPM or logTPM)?
    - The size of plots should be increased for better reader visibility.
    - It should be acknowledged in the main text that there are outliers in the low expression group that have high IHC classification, and that this is particularly evident for MCM4 and TYMS.
- Results
  - Page 12, Lines 156-165
    - It could be beneficial to report the log rank p-values for the IHC cut-offs here and acknowledge the cut-off for TYMS was not statistically significant.
  - Page 12, Line 162
    - Could the authors please clarify what >2p refers to? Is this positive staining with an intensity greater than 2?
  - Page 14
    - Were there any bias in between the IHC discovery and validation cohort that may account for why the gene expressions were only associated with gender and stage in the validation cohort?
      - Can the authors confirm that the IHC discovery cohort included advanced stages?
      - Additionally, was there an overall balance of male and female patients in both cohorts?
- Discussion
  - Microarray datasets
    - It may be useful to include general information about the microarrays to show the selected datasets capture a range of stage (early, advanced), growth patterns (mixed subtype, invasive) and ethnicities (Japanese, Caucasian).
  - Page 19, Line 273
    - The authors mention that the correlation between gene expression levels and IHC classifications was demonstrated for both IHC cohorts. However, this data is only shown for the IHC validation cohort (Supplementary Figure 7). Either amend this statement or add a supplementary figure for the IHC discovery cohort.

**Additional comments**

- Could the authors please provide a rationale for why they have included squamous samples in the IHC discovery cohort when gene expression-based discovery and validation was done only with lung adenocarcinoma cases?
  - This data doesn’t align with the rest of the paper and the authors could consider omitting this data.
- Typographical or grammatical errors
  - Page 19, Line 267
    - Reproducibility across IHC **remains challenging**
  - Page 19, Line 270
    - Might **have also** been considered
  - Page 19, line 276
    - Please use consistent italicisation for gene names
      - Italics are used here but not elsewhere in the manuscript
