# Supplementary material for: Gene expression-based identification of prognostic markers in lung adenocarcinoma
Source: PLoS One. 2025 May 7;20(5):e0310232. doi: 10.1371/journal.pone.0310232 (PMC12057878; doi:10.1371/journal.pone.0310232)
Supplement: Attachment — Submitted filename: Response to Reviewers.docx [file pone.0310232.s013.docx]

Manuscript PONE-D-24-37221 (Salomonsson et al.)

**Detailed point-by-point response to the reviewers’ comments**

Firstly, we thank both reviewers for their time and effort, for rigorous and in-depth discussion of our results, and for constructive suggestions on how to improve and clarify our manuscript "Gene expression-based identification of prognostic markers in lung adenocarcinoma". We also thank the editors for the possibility to revise our work based on the reviewers’ comments.

Please find our point-by-point response below. Reviewer’s comments are shown in bold font and our responses are written in regular text. Text in italics corresponds to our revised version of the manuscript and these changes are also marked in detail in a separate file (manuscript with word track changes).

**Reviewer #1:**

**The authors use publicly available microarray datasets to identify genes whose expression is associated with OS differences in lung adenocarcinoma and then tests clinical utility of select genes using IHC in Swedish cohorts. While correct statistical analysis was used and all conclusions are supported by the data, I had some minor concerns where further details or clarification are needed.**

**Major**

- **Methods**
- **Page 5, Figure 1**
  - **The authors may need to perform QC and additional filtering on microarray datasets. For example, Shedden et al. only retained array data for 442 of the 486 samples due to poor array quality (11 samples) or due to violations of clinical/pathological data (33 samples).**

The reviewer raises an important remark concerning usage of high-dimensional data (in-house generated or from public repositories). It is correct that Shedden et al. removed a set of cases from the final analysis. In the original data we downloaded raw data CEL files from the Shedden et al. publication (from 2008), using the link in the paper. In the original publication there was sample annotations for 486 samples. This link is now, however, non-functioning. Instead, the Shedden cohort has been placed in GEO ([GSE68465](https://www.ncbi.nlm.nih.gov/geo/query/acc.cgi?acc=GSE68465)**, deposited 7-years after the original publication**). In this repository there are only 462 assays included. In our analysis we used 444 CEL normalized files (samples) based on exclusion of the “poor” quality samples from what we could match to the original Shedden data. We performed the normalization ourselves.

From the GSE68465 deposit we have now crossmapped the file “DCLungStudy_Not_In_Study_Reasons.xlsx” versus our 444 actually used samples. 2 samples in the exclude file cannot be accounted for: “CL20041119193AA” and “NCI_CONTROL5_U133A”. As we cannot perform a trace back to the original Shedden deposit this limits the ability to identify why there is a missmatch between what the authors deposited 2008 and 2015 for download with respect to assays to exclude.

Moreover, in GSE68465 there is a file called “DCLungStudy_dChip-Processed_microarray_data.xlsx” holding apparently normalized CEL data, with a sheet “NotInStudy”. This sheet only seems to list 33 unique sample names. In the same file the depositing authors do not even list sample names for the sites HLM and MICH, making it difficult to link this normalized data correctly to sample annotations.

In response to the comment, we have updated Figure 1 with 444 samples used for the Shedden et al. cohort instead of the (by mistake on our side) stated 486. Thus, 444 is the actual number used.

While the discrepancy is irritating, it should also be noted that Shedden et al. reported about site specific bias, i.e. batch effects associated with the site where the data was generated, which was not completely removed in the study analyses. As outlined in previous publications by us, using the 444 samples we employed MAS5 site normalisation to downloaded CEL files (i.e. we reperformed the normalisation ourselves) similar to Shedden et al. to account for this. Importantly, while single cohort based studies could be highly affected by including bias / assay qualities the current study uses a systematic approach of multiple discovery and validation cohorts to derive target genes, including also different microarray platforms. If anything, we would most likely lose prognostic significance by including poor quality samples in a cohort, or alternatively find significance (genes) that would not validate in other cohorts. Notably, it is most likely that the variation between public cohorts with respect to outcome (e.g. “intrinsic” risk of a cohort due to its patient composition, treatment, and potentially also ethnicity) would by far outweigh the effect within a cohort of including cases with potentially poorter technical qualities. As such, the current study with its multiple discovery and validation cohorts comprising of different technical platforms, patient ethnicities, cohort compositions etc should support “solid” gene expression candidate targets.

We thank the reviewer for bringing attention to the discrepancies. In addition to updating Figure 1, we have made consequent revisions of the numbers stated in the main manuscript:

Lines 30-31: *In brief, we explored six different publicly available gene expression data sets, in total comprising* ***1,125*** *lung adenocarcinomas,*

Lines 36-37: *Samples with AC histology (n =* ***1,125****) were chosen for further comparisons of the gene expression data,*

- **Additionally, the number of samples don’t always align with those reported in the original publications. Why did the authors only include 102 of the 193 samples from Chitale et al?**

With respect to Chitale et al., this cohort comprises of 2 array subset, one performed on Affymetrix U133A arrays, and one on U133 2plus arrays (n=102). We only used the latter U133 2plus subset of the patients as outlined in previous studies by our group (e.g PMID: [32745259](https://pubmed.ncbi.nlm.nih.gov/32745259/)). For technical reasons we did not want to mix these two array types in a single cohort analysis as there would be batch effects based on platform (likely larger than the site specific batch effects in the Shedden cohort performed on the same array platform).

In the revised Figure 1 legend (page 4), we have clarified that we only use U133 2plus assayed samples from Chitale et al. We thank the reviewer for highlighting this need of clarification, improving the new version of our manuscript.

- **Similarly, how did you end up with 226 samples from Yamauchi et al? Was only the NCC-Tokyo cohort used from this publication?**
  - **The earliest publication associated with the GSE31210 dataset is Okayama et al. Cancer Research (2012) and they clearly state microarray data was generated for 226 cases. I would recommend either citing Okayama et al or both references for this dataset.**

We thank the reviewer for bringing our attention to this. We have changed the reference in text and figure from Yamauchi et al. to Okayama et al.

- **For improved clarity, it may be worthwhile to list the GEO accession ID for each of the microarray datasets.**

We thank the reviewer for this suggestion and we have now included the respective GEO no. or equivalent accession no. for the publically available datasets presented within Figure 1 of our revised manuscript. However, the Chitale dataset can no longer be reached via any viable link and is therefore best accessed from the corresponding author of the original publication (Chitale et al. 2009).

**Page 9, Line 112**

- **Could the authors please provide the rationale for including tertile split survival groups in addition to the median split survival groups?**
  - **It is not advised to use multiple different cutpoints unless it can be justified as it can appear to be a data “fishing” exercise, especially when the median cutpoint has been used consistently for all other gene expression analyses.**

We thank the reviewer for adequately pointing out difficulties in how groups with different prognosis are best separated from each other in this paper`s various survival analyses. We chose to allow division of the samples into either two or three equally sized groups only when we specifically wanted to investigate the prognostic value of the *Ki67*, *MCM4*, and *TYMS* gene expression levels within one of the IHC cohorts in our study. We motivate this by the fact that this analysis was not part of our actual proof-of-concept, i.e. our gene expression-based strategy for identification of prognostic markers and subsequent evaluation by IHC, on which we build this manuscript. Instead, we performed this part of the work to confirm a similar pattern on the gene expression level as on the protein level for the included samples.

In response to the comment, we have removed the tertile split survival analyses and instead used only the median cut point, as suggested by the reviewer. Accordingly, in the revised version of our article, supplementary figure 8 has been updated and formulations about dividing the samples into three groups have been omitted from the text in the main manuscript, i.e. both in Materials and Methods (Please see Word Track Changes, page 7, line 115-117) and in Results (two small text deletions on page 14, line 246 and 251, within the section Gene expression of Ki67, MCM4 and TYMS in the IHC validation cohort). We thank the reviewer for this improvement of our manuscript.

- **Results**
  - **Page 15**
    - **Could the authors please include forest plots for the Cox proportional models shown in Figure 4? This would confirm the relationship between established prognostic factors (such as stage) and OS are as expected in this cohort.**

We thank the reviewer for this suggestion about presenting the relationship between established prognostic factors and OS in our cohort. In response to the reviewer´s comment and in order to demonstrate that the material behaves as expected, we have now added a supplementary table where the hazard ratios for the various factors are reported. In the revised version of the manuscript, we refer to this new Suppl . Table 2 in the section where we already mention the multivariate model (page 15, line 218-219): *However, these results did not remain statistically significant in the multivariable model adjusted for stage, growth pattern, age, gender, smoking, WHO performance status, and adjuvant treatment (HR 1.29, 95% CI 0.68-2.45, Supplementary Table 2).*

- **Page 17, Line 218**
  - **I would recommend acknowledging that gene expression analysis of the IHC validation cohort was carried out using RNAseq rather than microarray as for the gene expression discovery and validation cohorts. This could also be mentioned in the discussion as technical differences between the platforms may have contributed to the results.**

We thank the reviewer for pointing out this need for clarification. As a consequence, we have added this information on page 13, line 236 and onwards. The new wording is as follows: *Gene expression levels of Ki67, MCM4, and TYMS, obtained by RNA sequencing analysis, were available for 104 AC cases in the IHC validation cohort.*

**Minor**

- **Figures**
  - **Page 11, Figure 2**
    - **Please update the figure legend to include details such as what the size of circles indicates.**

We thank the reviewer for pointing out this need for clarification. Correlation plots were performed using the corrplot R package. Here, the area of the circles show the absolute value of corresponding correlation coefficients. In the revised manuscript we have updated the Figure 2 legend (page 9) with this information (Please see the file “Revised Manuscript with Track Changes”).

- **Page 28, Supplementary Figure 3**
  - **Please add scale bars to all IHC images. Additionally, so that figure can be understood on its own, the figure legend should include information about the criteria for positive staining (e.g. nuclear or nuclear and cytoplasmic).**

We thank the reviewer for these suggestions and we have revised suppl. fig. 3 accordingly. Thus, for further clarity, a scale bar has been added to the figure and, in addition, we have complemented the figure legend with the requested information. In the revised version, the figure legend is as follows:

*Representative microscopic images of stainings. For Ki67 and MCM4, nuclear staining was considered positive. For TYMS, cytoplasmic or nuclear staining were considered positive, although only cells with visible nuclei were counted. (A) Low expression of Ki67. (B) High expression of Ki67. (C) Low expression of MCM4. (D) High expression of MCM4. (E) Low expression of TYMS. (F) High expression of TYMS.*

- **Page 31, Supplementary Figure 7**
  - **Can the authors confirm what expression units for gene expression levels (e.g. logCPM or logTPM)?**

We thank the reviewer for bringing our attention to this need for clarification. The publicly available data for this cohort from GEO (GSE81089) includes FPKM data based on Cufflinks estimation. We have revised the figure legend accordingly and in the revised version it will read as follows:

*The association between gene expression levels (y-axis, logFPKM) and immunohistochemical classification (x-axis, low or high expression) for the three markers in the IHC validation cohort.*

- **The size of plots should be increased for better reader visibility.**

Thank you for pointing this out. We have increased the figure sizes in the revised version of the supplementary information (S7 Fig).

- **It should be acknowledged in the main text that there are outliers in the low expression group that have high IHC classification, and that this is particularly evident for MCM4 and TYMS.**

We thank the reviewer for bringing our attention to this need for clarification. Accordingly, we have acknowledged this information in the main text (page 13):

*For all three markers, an association between gene expression levels and IHC classification (low or high expression) could be observed (Wilcoxon test, P < 0.01 all three tests). However, and most evident for MCM4 and TYMS, there were also cases with high gene expression that were classified as low expressors by IHC (Supplementary Figure 7).*

- **Results**
  - **Page 12, Lines 156-165**
    - **It could be beneficial to report the log rank p-values for the IHC cut-offs here and acknowledge the cut-off for TYMS was not statistically significant.**
    - **Could the authors please clarify what >2p refers to? Is this positive staining with an intensity greater than 2?**

For further clarity, and in accordance with the reiewer´s suggestions, we have added more detailed reports on the log rank p-values for the cut-offs in the main text on page 10-11, line 161-180 (see below). We also further explained the score (for combining fraction and intensity in the TYMS evaluation) in Materials and Methods (page 7, line 104): *For TYMS, in addition to the recorded fraction of positive tumor cells, the staining intensity was scored as 0 (negative), 1 (mild), 2 (moderate), or 3 (strong) and a final score was constructed by multiplying these two parameters, thus ranging from 0-15 points*.

The revised text on page 10-11 now reads as follows:

*For Ki67, a cut-off of >10% positive tumor cells most clearly identified prognostic groups in the OS analysis (log rank p-value 0.014) among the AC cases and was therefore chosen for identification of samples with a low or high expression (Figure 3A). By applying this cut-off, 74 AC cases (56%) were classified as having a high Ki67 protein expression. A cut-off of >75% positive tumor cells was selected for MCM4 among the AC cases in the OS analysis (log rank p-value 0.0005), which resulted in 15 cases (12%) identified as having a high MCM4 expression (Figure 3B). For TYMS, a score (obtained by multiplying fraction and intensity, as further explained in Materials and Methods) of >2 points was chosen for identification of AC samples with a high TYMS expression, which resulted in 19 cases (16%) classified as having a high expression of TYMS (Figure 3C) but was not statistically significant in the OS analysis (log rank p-value 0.055). The prognostic value of these cut-offs for Ki67, MCM4, and TYMS in the RFI-analysis are shown in Supplementary Figure 4.*

- **Page 14**
  - **Were there any bias in between the IHC discovery and validation cohort that may account for why the gene expressions were only associated with gender and stage in the validation cohort?**
    - **Can the authors confirm that the IHC discovery cohort included advanced stages?**
    - **Additionally, was there an overall balance of male and female patients in both cohorts?**

We thank the reviewer for pointing out this need for clarification. To achieve this, we have added information regarding bias in the main text (page 5, line 68-69):

*Patient characteristics and clinicopathological data were described previously [20]. The two IHC cohorts were balanced regarding age, gender, smoking, stage, and treatment.*

- **Discussion**
  - **Microarray datasets**
    - **It may be useful to include general information about the microarrays to show the selected datasets capture a range of stage (early, advanced), growth patterns (mixed subtype, invasive) and ethnicities (Japanese, Caucasian).**

We thank the reviewer for this suggestion. Accordingly, we have included more information about the publically available datasets in the discussion section (page 14, line 260-264):

*As a proof-of-concept, we identified markers with prognostic impact in lung adenocarcinoma through a gene expression-based, multi-cohort discovery and validation strategy. Based on global gene expression profiling array data sets generated from original analyses of early (operable stage I-III) lung adenocarcinomas in North American, European, and Japanese patient populations, we were able to associate the expression of 19 genes to survival in a total of six independent studies, published in reputable journals [10-15]. We also selected markers identified by this strategy for further evaluation by IHC, a method more adapted to the current clinical setting, thus underlining a potential future clinical utility.*

**Page 19, Line 273**

- **The authors mention that the correlation between gene expression levels and IHC classifications was demonstrated for both IHC cohorts. However, this data is only shown for the IHC validation cohort (Supplementary Figure 7). Either amend this statement or add a supplementary figure for the IHC discovery cohort.**

We thank the reviewer for pointing this out. We have corrected the text (page 16, line 303) in accordance with this comment:

*The robustness of the three selected markers was demonstrated through a clear correlation between gene expression levels and IHC classification in the IHC validation cohort.* (i.e. the wording “in both cohorts” has been omitted.)

**Additional comments**

- **Could the authors please provide a rationale for why they have included squamous samples in the IHC discovery cohort when gene expression-based discovery and validation was done only with lung adenocarcinoma cases? This data doesn’t align with the rest of the paper and the authors could consider omitting this data.**

We thank the reviewer for bringing our attention to the risk of confusion based on us presenting data also from sqamous cell samples in the IHC cohort. Accordingly, we decided to follow the reviewer´s recommendation and have omitted this data. As a consequence, involved sections within the manuscript have been revised (Please see the separate Word Track Changes file). Deleted texts:

Page 5, line 64: “and 69 SqCC”

Page 7, line 107: “Prognostic analyses were performed separately on AC and SqCC”

Page 10, line 161 and onwards: “For SqCC, 68 cases ……………………… …………………… …analyses were performed on AC and SqCC separately.”

Page 11, line 181 and onwards: “For SqCC, no prognostically meaningful ……………………………………….. evaluated in the IHC validation cohort.”

Page 16, line 316-326: “Only AC cases were included in…..…………………… …………find meaningful subgroups based on these markers.”

Page 17, line 357-358: “Also, in the IHC-cohorts, the SqCC cases were few and results should be interpreted with care.”

- **Typographical or grammatical errors**
  - **Page 19, Line 267**
    - **Reproducibility across IHC remains challenging**

The error has been changed (page 15, line 295) in accordance with the reviewer´s comment. Thank you for pointing it out.

- **Page 19, Line 270**
  - **Might have also been considered**

The error has been changed (page 16, line 300) in accordance with the reviewer´s comment. We thank the reviewer for pointing this out.

- **Page 19, line 276**
  - **Please use consistent italicisation for gene names**
    - **Italics are used here but not elsewhere in the manuscript**

In accordance with the reviewer´s comment, we have corrected this in the abstract and in the Discussion section (page 15, line 287), where (by our mistake) italics were not used in our original version. Thank you for pointing this out.

**Reviewer #2:**

**The research paper presents a comprehensive study on non-small cell lung cancer (NSCLC) data sets, where, via the means of finding strong statistical associations between gene expression and cancer patient overall survival data, the authors have extracted a list of genes serving as potential prognostic markers for adenocarcinoma-type lung cancer. These markers’ prognostic ability was then assessed on a protein level via immunohistochemistry (IHC), where protein expression was evaluated and its association with overall survival and recurrence-free interval in two patient cohorts was statistically tested.
Overall, the manuscript is well written and shows a strong methodology for predicting prognostic markers for NSCLC using gene expression data. The statistical analyses applied were appropriate for the research question and tested the data stringently. The negative findings (i.e. the results that did not reach statistical significance) were addressed in the discussion section, and the limitations of the study were acknowledged.
High-quality data sets were used for the discovery of prognostic markers and yielded excellent results, revealing a set of correlated genes involved in tumour proliferation. Further IHC experiments were designed with care and were able to demonstrate the promising potential of gene expression-based prognostic research, confirming some, but not all, of the associations with patient survival on the level of both gene and protein expression. Although the IHC validation data set has revealed few statistically significant results, using different expression level cut-offs, as noted by the authors, could potentially result in more positive findings. It also appears that multivariate Cox proportional hazards regression models were another high-impact factor that lowered the findings’ significance levels. It is possible that the size of the data set was too small relative to the number of predictor variables used, resulting in higher p-values; however, this may be subjective, as no standard cut-off for events per variable (EPV) has been defined in current literature.**

**With regard to manuscript revisions, I only have suggestions concerning a few minor points. I would recommend the authors to consider substituting the term “correlation” where it is not in the context of the actual correlation analyses. For example, on page 4, lines 31-32: “… to identify and validate markers with consistent correlation to overall survival (OS)”. The term “correlation”, although semantically correct, may be misleading here, as the relationship between OS and marker gene expression was established via a log-rank test. It would be better to replace the term “correlation” with “association” as it was already done in several parts of the text and within Figure 1.
Several minor typos and grammatical errors were spotted, therefore additional proofreading is recommended.**

**After these minor revisions are made, I recommend this manuscript for publication.**

We thank the reviewer for this positive discussion of our paper and for constructive suggestions for improvements.

In accordance with the reviewer´s comments, we have replaced “correlated/correlation” with “associated/association” in the following places in the main text:

Page 4, line 31-32

Page 13, line 239

Page 15, line 275

**Journal requirements**

Furthermore, in response to the academic editor´s comment on validation method for the antibodies used, we have added information of the control tissue used for our internal validation in Supplementary Table 1 and we thank the editor for pointing out this need for clarification.

In addition to our responses to the reviewer´s comments above, we submit a revised manuscript, the revised manuscript with track changes, and all supporting information as separate files. In addition, the manuscript figures 1-4 have been uploaded in PACE. All relevant data are either within the manuscript and its Supporting Information files or can be accessed via the accession identification numbers of the original publications. All authors have declared that no competing interests exist.

Finally, we thank the editor and the reviewers for your time and effort and for the very constructive suggestions that we believe have improved our manuscript.

Best regards,

Maria Planck, MD, PhD

Senior Consultant and Associate Professor

Skane University Hospital and Lund University
